# Optimizing Mask R-CNN for enhanced quinoa panicle detection and segmentation in precision agriculture

**DOI:** 10.3389/fpls.2025.1472688

**Published:** 2025-06-02

**Authors:** Manal El Akrouchi, Manal Mhada, Dachena Romain Gracia, Malcolm J. Hawkesford, Bruno Gérard

**Affiliations:** ^1^ College of Agriculture and Environmental Sciences, University Mohammed VI Polytechnic (UM6P), Ben Guerir, Morocco; ^2^ School of Collective Intelligence, University Mohammed VI Polytechnic (UM6P), Rabat, Morocco; ^3^ Sustainable Soils and Crops Department, Rothamsted Research, Harpenden, Hertfordshire, United Kingdom

**Keywords:** Mask R-CNN, instance segmentation, quinoa, precision agriculture, deep learning

## Abstract

Quinoa is a resilient, nutrient-rich crop with strong potential for cultivation in marginal environments, yet it remains underutilized and under-researched, particularly in the context of automated yield estimation. In this study, we introduce a novel deep learning approach for quinoa panicle detection and counting using instance segmentation via Mask R-CNN, enhanced with an EfficientNet-B7 backbone and Mish activation function. We conducted a comparative analysis of various backbone architectures, and our improved model demonstrated superior performance in accurately detecting and segmenting individual panicles. This instance-level detection enables more precise yield estimation and offers a significant advancement over traditional methods. To the best of our knowledge, this is the first application of instance segmentation for quinoa panicle analysis, highlighting the potential of advanced deep learning techniques in agricultural monitoring and contributing valuable benchmarks for future AI-driven research in quinoa cultivation.

## 1 Introduction

Quinoa, indigenous to the Andean regions of South America, is gaining worldwide recognition for its exceptional nutritional value. As a “nutrient-dense food”, it is an outstanding choice for health-conscious consumers, rich in proteins, dietary fiber, and essential vitamins and minerals (Pathan and Siddiqui, 2022). Notably, quinoa is gluten-free and an excellent source of proteins, containing all nine essential amino acids, which are classified as essential because mammals cannot synthesize them. Consequently, these amino acids must be obtained through dietary sources to support various physiological functions. Quinoa is therefore recommended for vegans, vegetarians, and gluten-intolerant individuals (Pathan and Siddiqui, 2022).

In addition to its impressive nutrient profile, quinoa is remarkably resilient to various stressors, including drought and salinity: some quinoa genotypes can tolerate soil salinity levels up to approximately 32 dS/m, whereas wheat experiences salinity tolerance at around 7.13 dS/m (Peterson and Murphy, 2015; Chaganti and Ganjegunte, 2022; Hussin et al., 2023; Ehtaiwesh et al., 2024; Gheisary et al., 2025), positioning quinoa as a promising candidate for global cultivation (Cai and Gao, 2020). Its robustness, coupled with its nutritional benefits, has positioned quinoa as a crop with significant potential to address global food security challenges (Jacobsen, 2003; Bazile et al., 2016b; Akhlaq et al., 2023). Although not yet as widely cultivated as staple crops like wheat or rice, quinoa has been introduced in over 100 countries ^1^ (Jacobsen et al., 2003; Jacobsen, 2003) and promoted by the FAO as a strategic crop to combat malnutrition and diversify diets. Its ability to grow in arid and saline soils positions it as a valuable alternative for millions (Adolf et al., 2013; Bazile et al., 2016a) living in regions unsuitable for conventional crops.

Despite quinoa’s growing global importance, several key aspects of its cultivation remain understudied, including pest tolerance, nutritional composition, yield prediction, and farm management strategies. Addressing these gaps is essential for optimizing quinoa production and enhancing its resilience in diverse agricultural settings. However, traditional farming practices still pose challenges, often leading to suboptimal yields due to inefficient crop management and limited mechanization. Additionally, quinoa’s morphological complexity, particularly the dense clustering and variability in panicle structure, makes manual phenotyping labor-intensive and error-prone, further complicating efforts to assess plant health and productivity accurately (Pathan and Siddiqui, 2022).

The potential of Artificial Intelligence (AI) in managing crops such as quinoa is vast. With advanced machine learning algorithms, such as instance segmentation methods, AI has the power to revolutionize crop management. By detecting panicles – flower-bearing structures of quinoa that develop into seed clusters, they vary in shape, size, and density across different genotypes and play a crucial role in determining crop yield– and analyzing weather patterns, soil conditions, and crop health, AI can accurately predict crop yield, empowering farmers to make informed decisions and increase productivity. Such predictions can significantly aid farmers in making informed decisions about irrigation, fertilization, and pest management (Mohr and Kühl, 2021). Furthermore, integrating AI into precision agriculture can automate labor-intensive research tasks (Alreshidi, 2019), such as counting the number of panicles, estimating their size, and even detecting early-stage crop diseases. Moreover, AI plays an essential role in improving breeding and phenotyping. These advancements will increase the efficiency of farming practices in addition to decisionmaking support for farmers and contribute to food security by maximizing the yield of nutritionally dense crops like quinoa (Sanders et al., 2021).

The development of AI technology in computer vision has opened up exciting new possibilities for practical applications. Instance segmentation is a challenging technique in computer vision that involves identifying and classifying individual objects within an image. It assigns a unique label to each object and is useful in applications such as autonomous driving. However, it is challenging due to the variety of object shapes and sizes, complex backgrounds, and partial occlusion (He et al., 2020). In agriculture, instance segmentation plays a vital role in precision farming, where it has been shown to improve farming practices through the use of technology (Yang et al., 2020). One of the notable techniques in instance segmentation is Mask R-CNN.

Mask R-CNN, a well-known Region-based Convolutional Neural Networks (R-CNN) variant, performs well in instance segmentation (He et al., 2018). Built on Faster R-CNN, a two-stage object detection model, Mask R-CNN extends it by adding a stage that generates pixel-level segmentation masks for each detected object. It offers a powerful tool for complex tasks by dividing images into regions of interest, classifying them, and generating precise masks for each instance (He et al., 2020). In agriculture, Mask R-CNN can be deployed to identify individual panicles in crops, count them, and estimate their size. This technology has the potential to help farmers predict yield with higher precision, detect early-stage crop diseases, and manage their farms more efficiently (Yang et al., 2020). For quinoa panicle detection, this enhancement is crucial because panicles often overlap, vary in shape, and are densely packed. Drawing a bounding box (as in Faster R-CNN) would not provide the necessary granularity for accurate panicle counting and segmentation. Instead, Mask R-CNN allows us to separate individual panicles even when they touch or overlap, making it the best choice for our application.

While Mask R-CNN holds promise, systematic studies are needed to explore the performance of different backbones within this architecture, particularly in an agricultural context (Yan et al., 2023a). As backbones play a crucial role in extracting image features, this study addresses this gap by comprehensively assessing various backbones within Mask R-CNN. The goal is to detect and count the quinoa panicles. The main objectives of this research paper are threefold:

- It seeks to evaluate the effectiveness of different backbones of Mask R-CNN in instance segmentation for detecting panicles in quinoa. The tested backbones were selected from CNN-based architectures (ResNet50, ResNet101, EfficientNet-B7) and transformer-based architectures (Vision Transformer, Swin Transformer).
- It examines the accuracy of these models in matching the detected panicles.
- It introduces a new methodology for detecting and segmenting panicles, which could prove valuable in yield estimation tasks in precision agriculture.

The results of this study will add to the current understanding of instance segmentation in agriculture but also aid practitioners in selecting a backbone for Mask R-CNN when creating models for panicle detection, thereby improving the efficacy of precision agriculture applications.

## 2 Background overview

### 2.1 Quinoa

Over the past decade, quinoa (*Chenopodium quinoa* Willd) has gained international recognition for its exceptional nutritional value and adaptability to diverse environments (Mhada et al., 2020). Its high protein content, rich amino acid profile, and resilience to abiotic stresses make it a promising crop for enhancing food security and reducing the environmental impact of agriculture (Khaitov et al., 2021). Additionally, integrating quinoa into cropping systems promotes agricultural diversification, offering a strategic approach to stabilizing yields under varying climatic conditions (Verma et al., 2017).

One of the key determinants of quinoa yield is the number of panicles, the flowering structures that bear seeds. Traditionally, panicle counting has been labor-intensive and time-consuming, requiring manual evaluation for phenotyping, which involves assessing observable plant traits influenced by genetic and environmental interactions. Automating this process using advanced computer vision techniques can significantly enhance efficiency, reduce human error, and enable large-scale phenotypic assessments.

Recent advancements in artificial intelligence (AI) and computer vision have provided powerful tools for automated crop monitoring and yield estimation. However, Quinoa panicle identification remains particularly challenging due to its diverse morphological characteristics. While quinoa panicles have been broadly classified into three categories (Bioversity International et al., 2013), many panicles exhibit intermediate or atypical structures that do not fit neatly into these classifications, posing difficulties for deep learning-based detection models.

**Figure 1:**
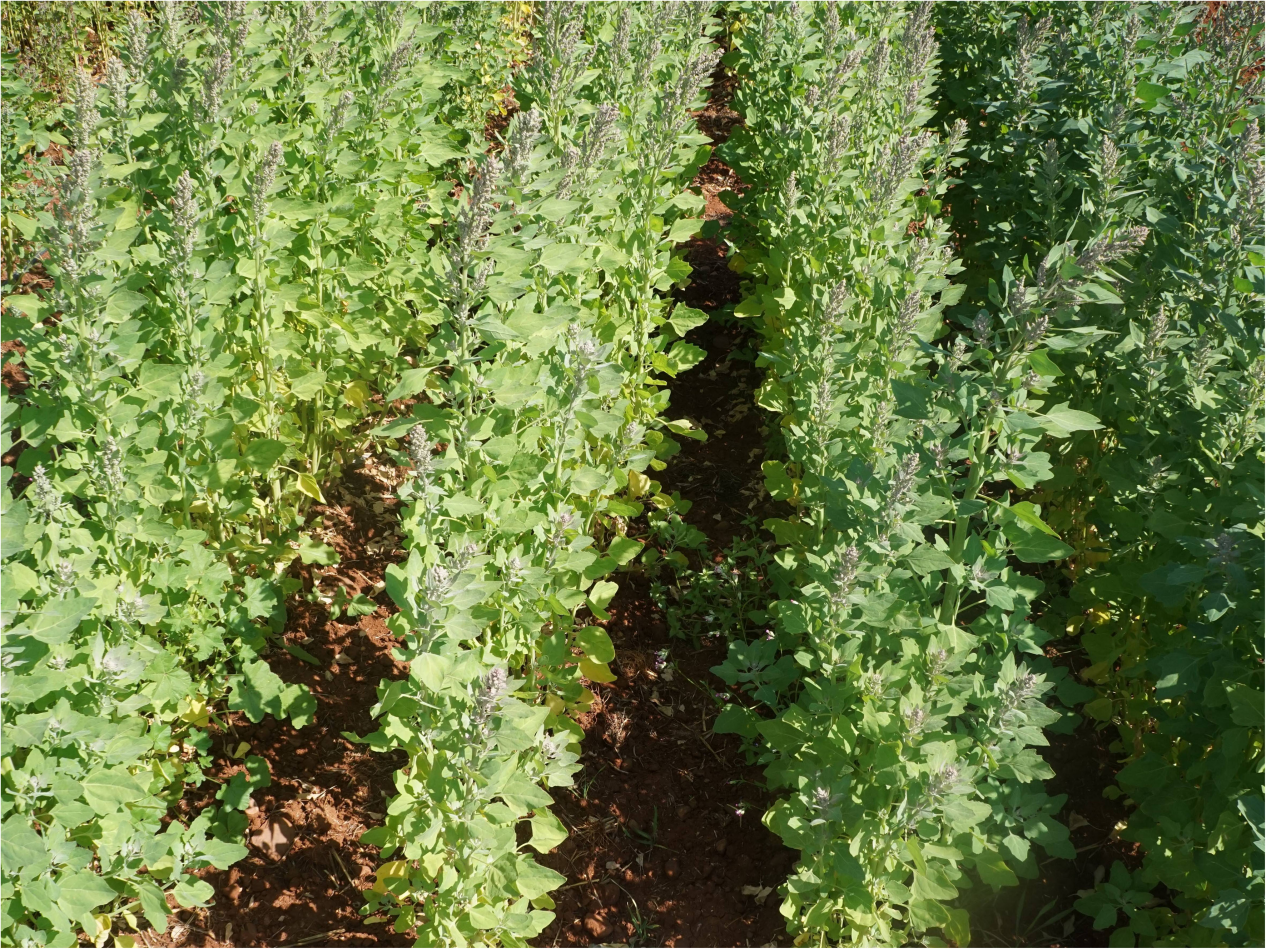
Example of in-field image of quinoa from the experiment used in this study.

Additionally, panicle density and color variation introduce further complexities in in-field image analysis. Densely clustered panicles can lead to occlusions, making it difficult to distinguish individual panicles, while variations in panicle color across different genotypes can cause misclassification or low segmentation accuracy. Some quinoa genotypes also exhibit unusual structural or pigmentation traits, which can confuse standard image processing algorithms, resulting in either false positives or missed detections. As illustrated in **Figure 1**, these idiosyncratic structures and colors can blend into the background or intertwine with other panicles, requiring advanced deep-learning techniques for accurate detection and quantification.

Given the complex structure of quinoa panicles and the limitations of conventional image-based detection methods, instance segmentation emerges as the most effective approach for precise identification and counting. Unlike traditional object detection methods that rely solely on bounding boxes, instance segmentation provides pixel-wise masks, allowing for precise differentiation of overlapping and densely clustered panicles. In quinoa, instance segmentation is essential for accurate panicle detection and counting, allowing researchers and breeders to analyze key yield-related traits such as panicle size, number, and density.

### 2.2 Instance segmentation

Object detection in computer vision involves identifying objects within an image by placing a bounding box around them to train the model. However, overlapping objects can be challenging to detect accurately with this method. To overcome this obstacle, instance segmentation, which provides a more precise annotation method, should be utilized.

Image segmentation is at the heart of many deep learning applications, including medical image analysis, automated driving, video surveillance, virtual and augmented reality, scene understanding, and robot perception. Image segmentation is the process of classifying each pixel in an image with the correct label so that pixels with the same label have specific characteristics.

Instance segmentation is a computer vision task for detecting and localizing an object in an image. Instance segmentation is a natural sequence of semantic segmentation and is one of the biggest challenges compared to other segmentation techniques. The goal of instance segmentation is to view objects of the same class divided into different instances. Its primary objective is to dissect digital visuals into distinct segments or regions, each representing a unique object or a specific segment of that object. Its dual capacity sets instance segmentation apart from other analogous methods: it classifies every pixel within a given image, pinpoints, and distinguishes individual object instances. Numerous methodologies have been innovated to achieve this intricate process, many of which harness the power of deep learning and convolutional neural networks. Various techniques of instance segmentation can be found in the literature, including Mask R-CNN (Region-based Convolutional Neural Networks) (He et al., 2018), YOLACT (You Only Look At CoefficienTs) (Bolya et al., 2019), SOLOv2 (Segmenting Objects by Locations) (Wang et al., 2020), and PointRend (Point-based Rendering) (Kirillov et al., 2020).

Deep learning-based instance segmentation is an active area of research, and new techniques continue to emerge as researchers strive to improve the speed, accuracy, and efficiency of these models. However, despite rapid development in this field, instance segmentation remains a challenging task, particularly for complex scenes with many overlapping objects, diverse object classes, and objects with intricate shapes. **Table 1** presents some research works done on different crops using instance segmentation.

**Table 1:** Summary of different studies using instance segmentation on various crops.

Study	Method used	Crop	Key findings	Limitations and gaps
Su et al (Su et al., 2020)	Mask R-CNN with ResNet-101+FPN	Wheat	AP50 values: 56.69% (detection) and 57.16% (segmentation) for wheat spikes	Limited to controlled conditions; not tested on quinoa
Shen et al (Shen et al., 2022)	Improved Mask RCNN with attention mechanism and Res-Net50+FPN	Grape	AP50 values: 85.60% (detection) and 87.10% (segmentation) for grape clusters	Focus on larger fruits; not applicable to small panicles
Jia et al (Jia et al., 2020)	Optimized Mask R-CNN	Apple	Effective segmentation of overlapped fruits with 92.3% accuracy	Focused on large fruits with distinct boundaries; not suitable for dense clusters like panicles
Kumar & Kukreja (Kumar and Kukreja, 2022)	Mask R-CNN	Wheat	Detection of mosaic virus on individual wheat leaves	Disease detection rather than yield estimation; different application focus
Kukreja et al (Kukreja et al., 2022)	Mask Scoring R-CNN	Wheat	Recognition of wheat aphid disease	Disease-specific application; not addressing yield prediction
Kumar et al (Kumar et al., 2023)	Mask R-CNN	Soybean	Leaf disease detection and segmentation	Limited to leaf analysis, not reproductive structures
Wang et al (Wang et al., 2021b)	Swin Transformer	Grape	Robust grape bunch detection in complex vineyard environments	Not evaluated on smaller, more complex structures like quinoa panicles
Yan et al (Yan et al., 2023b)	Transformer-based instance segmentation	Pumpkin	Grasping and cutting points detection for harvesting	Focused on harvesting logistics rather than yield estimation
Mache-fer et al (Machefer et al., 2020)	Mask R-CNN	Potato	Plant counting and sizing from UAV imagery	Aerial perspective limitations; not addressing ground-level detailed panicle detection

### 2.3 Mask R-CNN

Accurately detecting quinoa panicles is challenging due to their variations in shape, dense clustering, and occlusions. Instance segmentation methods address these challenges by distinguishing individual panicles within an image. Among the primary approaches, single-shot instance segmentation (e.g., YOLACT, SOLOv2) offers speed but lacks precision in separating overlapping panicles. Transformer-based methods (e.g., DETR, MaskFormer) excel at contextual reasoning but require extensive labeled datasets and high computational power, making them less practical for our study.

Given these constraints, we selected Mask R-CNN as the most suitable method because it generates pixel-level masks for each panicle, ensuring precise segmentation even in complex field conditions. Unlike single-shot methods, it effectively separates overlapping panicles, and compared to transformer-based approaches, it offers a balance of accuracy and computational efficiency.

Mask R-CNN is an advanced object detection and instance segmentation model that builds upon the Faster R-CNN architecture (He et al., 2018). Faster R-CNN is a two-stage object detection model that utilizes a Region Proposal Network (RPN) to generate high-quality region proposals, which are then used by the Fast R-CNN network for object detection (Ren et al., 2017). The RPN is a fully convolutional network that predicts object bounds and objectness scores at each position (Ren et al., 2017). Mask R-CNN extends this framework by adding a third stage, for instance, segmentation, to generate pixel-level masks for each detected object (He et al., 2020).

In agriculture, Mask R-CNN has been applied to various applications. One such application is crop monitoring and yield estimation. Mask R-CNN can detect and segment individual crops in aerial or satellite images, accurately estimating crop yield and health (Machefer et al., 2020). This information can be valuable for farmers in optimizing their agricultural practices and resource allocation. Another application is weed detection and management. Mask R-CNN can be trained to identify and segment weeds in agricultural fields, enabling targeted and precise weed control measures. This can help reduce the use of herbicides and minimize their environmental impact. Mask R-CNN has also been very useful in disease detection in crops. Using computer vision and especially instance segmentation to detect and recognize diseases in various crops is extremely important to prevent potential risks and losses. For example (Wen-Hao et al., 2020), evaluated Mask R-CNN to detect Fusarium Head Blight in wheat images.

### 2.4 Backbone networks

Extracting features is a crucial step in data analysis. Statistical algorithms and filters were initially used to extract features from input data for subsequent processing. However, with the advent of the machine and deep learning techniques, neural networks have revolutionized the process by providing improved performance and the ability to process larger volumes of data (Pietikäinen and Silven, 2022). With the development of convolutional neural networks (CNNs), it has become possible to work with large-scale data sizes and use them for feature extraction.

Choosing a CNN network for feature extraction or other parts of a deep learning model is not random. It requires careful consideration and analysis (Zhou et al., 2022). So, the implementation of such a model can be related to the target task as well as the complexity of it. These networks are used now for feature extraction or at the beginning of any DL model and its named backbones. A backbone is the recognized architecture or network used for feature extraction which has been trained in many other tasks previously with demonstrated effectiveness. This section will cover the most commonly used backbones for feature extraction suitable for the Mask R-CNN model.

#### 2.4.1 Resnet backbones

The ResNet (Residual Network) family is a powerful deep-learning architecture widely used in computer vision. Developed by Kaiming He and colleagues in 2015 (He et al., 2016), ResNet introduced a key innovation called “residual blocks,” which help train very deep neural networks more effectively. Normally, when a network becomes too deep, it struggles to learn properly due to a problem called the vanishing gradient, where important information fades as it moves through layers. ResNet solves this by making the network focus on learning the difference (“residual”) between the input and the expected output, rather than trying to learn everything from scratch. This clever technique allows ResNet models to train deeper networks without losing accuracy, making them highly effective for image recognition and object detection tasks.

These residual connections, also termed “skip connections,” bypass one or more layers and add the output from the previous layer to the output of subsequent layers. This approach enhances gradient flow through the network, enabling the training of much deeper networks than was previously feasible. The original ResNet paper demonstrated architectures with depths of up to 152 layers, shattering previous benchmarks on ImageNet and COCO datasets.

In ResNet models, all convolutional layers apply the same 3 × 3 convolutional window, and the number of filters increases with network depth, from 64 to 512 (for ResNet-18 and ResNet-34), from 64 to 2048 (for ResNet-50, ResNet-101, and ResNet-152). For all models, there is only one max-pooling layer with a pooling size of 3 × 3, and a stride of 2 is applied after the first layer. Therefore, reducing the input’s resolution during training is severely limited. At the end of all models, the average pooling layer is applied to replace the fully connected layers. This replacement has several advantages. First, this layer has no parameters to optimize, so it helps reduce model complexity. Second, this layer is more native in enforcing the correspondences between feature maps and categories. In this study, ResNet50 and ResNet101 will be covered.

#### 2.4.2 Transformers backbones

Transformers have become famous for backbone architectures in various domains, including natural language processing and computer vision. In natural language processing, the RoBERTa model, proposed by (Liu et al., 2019), has demonstrated robust performance by optimizing the BERT pretraining approach. The authors conducted a replication study of BERT pretraining and found that hyperparameter choices and training data size significantly impact the final results.

In computer vision, transformers have also been utilized as backbone architectures (Wang et al., 2022b). studied two families of backbones for semantic segmentation: convolutional neural networks (CNNs) and vision transformers. They used the original ResNet-50 as a CNN backbone and compared it to the commonly used inception stem (Qiang et al., 2023). introduced the SeaFormer framework, a squeeze-enhanced axial transformer explicitly designed for mobile semantic segmentation. They demonstrated superior performance on datasets such as ADE20K and Cityscapes, surpassing mobile-friendly rivals and transformer-based segmentation models. Furthermore, SeaFormer showed potential as a versatile, mobile-friendly backbone for image classification.

In the context of dense prediction in computer vision (Ranftl et al., 2021), proposed using vision transformers as the backbone in an encoder-decoder structure. They showed how the representations produced by the vision transformers can be effectively transformed into dense predictions, leading to state-of-the-art results in dense prediction tasks.

Transformers have emerged as a versatile and powerful choice for backbone architectures in computer vision. Their ability to capture long-range dependencies and learn complex patterns has significantly advanced various tasks. This study selected two widely used transformers in computer vision: ViT (VisionTransformer) and Swin. The definition of these backbones is presented:

ViT (Vision Transformer):

The Vision Transformer (ViT) (Dosovitskiy et al., 2021) represents a notable shift in the approach to visual recognition tasks, moving away from the long-standing dominance of convolutional neural networks (CNNs) toward the realm of transformers, which have been immensely successful in natural language processing. Instead of relying on convolutions to process image data locally, the ViT backbone takes an image, splits it into a sequence of fixed-size non-overlapping patches, linearly embeds these patches into flat vectors, and then processes them in a sequence just like words in a sentence. Positional embeddings are added to the patch embeddings to provide positional information, which is inherently absent when using transformers. The transformer backbone then processes this sequence through self-attention mechanisms and feed-forward networks, enabling it to weigh the importance of different patches. The result is a model that can capture an image’s local and global patterns.

Swin Transformer:

The Swin Transformer is an innovative vision transformer model that utilizes a unique “shifted windows” method to process images (Liu et al., 2021). Unlike traditional transformer models that divide images into non-overlapping patches of fixed size, the Swin Transformer divides images into overlapping patches that are shifted by a certain amount. This technique enables the model to capture local and global information in the image and handle large objects that may span multiple patches. Furthermore, the Swin Transformer employs a hierarchical architecture to process the image at various scales, allowing it to capture fine-grained details and high-level contextual information. As a result of these features, the Swin Transformer has achieved state-of-the-art performance on benchmark datasets such as ImageNet and COCO object detection.

The success of the Swin Transformer in image recognition tasks demonstrates the potential for Transformer-based models to be applied in the vision domain (Cao et al., 2021). It has been used as a backbone in various applications, including medical image segmentation (Cao et al., 2021), music classification (Zhao et al., 2022), image denoising (Fan et al., 2022), grape bunch detection (Wang et al., 2021a), lettuce browning prediction (Wang et al., 2022a), optical chemical structure recognition (Xu et al., 2022), and single image dehazing (Yang et al., 2022). These applications highlight the versatility and effectiveness of the Swin Transformer in different domains and tasks.

#### 2.4.3 EfficientNet backbones

EfficientNet is a family of convolutional neural network (CNN) models designed to balance accuracy and efficiency by scaling the network dimensions of depth, width, and resolution (Tan and Le, 2019). The EfficientNet models have achieved state-of-the-art performance on various computer vision tasks, including image classification, object detection, and semantic segmentation (Tan and Le, 2019; Marques et al., 2020).

The EfficientNet models are built on top of the MobileNetV2 architecture, which utilizes inverted residuals and linear bottlenecks to improve the performance of mobile models (Sandler et al., 2018). The MobileNetV2 architecture incorporates a novel framework called SSDLite for object detection and a reduced form of DeepLabv3 (Chen et al., 2018) called Mobile DeepLabv3 for semantic segmentation (Sandler et al., 2018).

**Figure 2:**
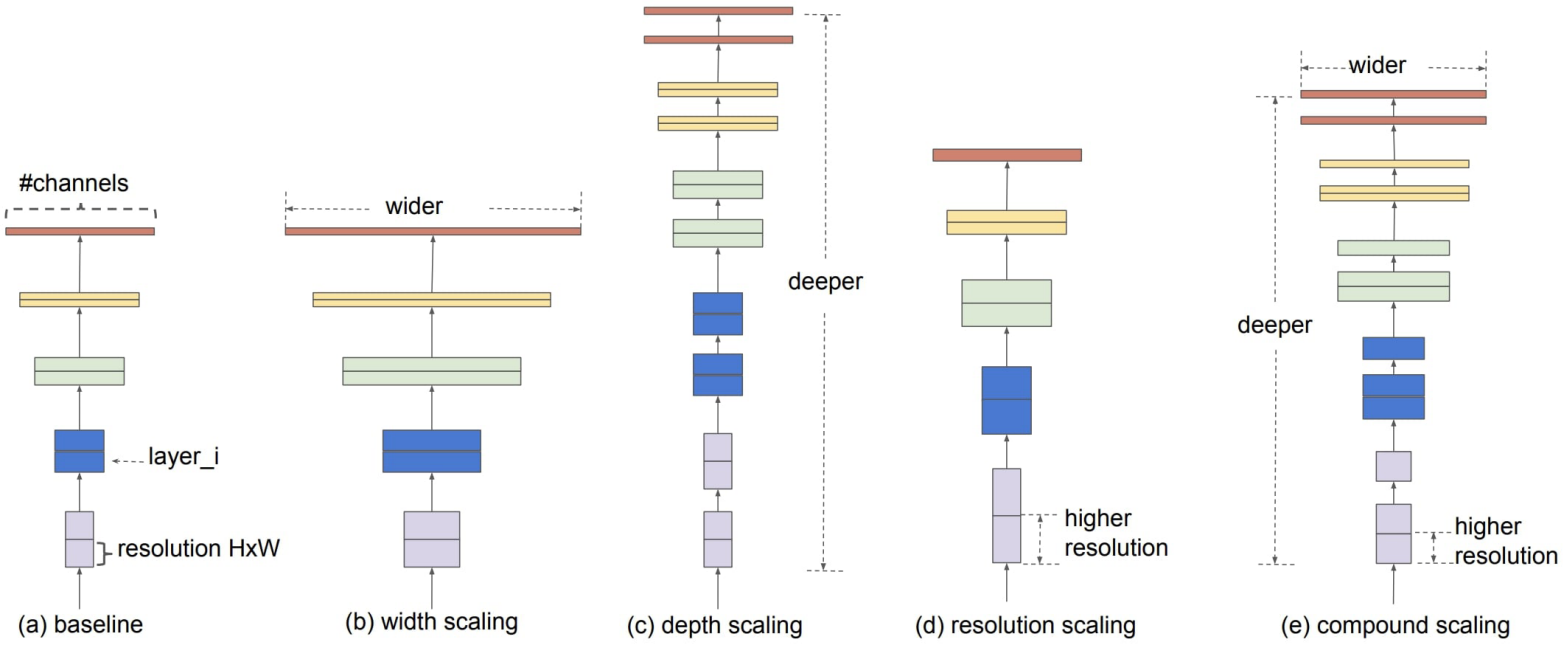
Model scaling (Tan and Le, 2019). **(a)** is a baseline network example; **(b–d)** are conventional scaling, increasing only one network width, depth, or resolution dimension. **(e)** is the compound scaling method, which scales all three dimensions at a fixed ratio in a uniform manner.

EfficientNet uses a technique called Compound Coefficients to scale models in a simple but effective way (Tan and Le, 2019). With compound scaling, each dimension is scaled uniformly by a fixed set of scaling coefficients rather than randomly scaling width, depth, or resolution. The Efficientnet authors developed seven models of different dimensions that surpassed the state-of-the-art accuracy of most convolutional neural networks with much better efficiency using compound scaling and AutoML. **Figure 2** shows the composite scaling method.

The EfficientNet-B0 architecture was developed using a multi-objective neural architecture search that optimizes accuracy and floating-point operations. Taking B0 as a baseline model, the authors (Tan and Le, 2019) developed an entire family of EfficientNets from B1 to B7, which achieved state-of-the-art accuracy on ImageNet while being very efficient to its competitors. Based on the concept of the Compound Coefficients mentioned earlier, depth, width, and resolution parameters can be modified to scale up the baseline network to obtain EfficientNet-b1 to b7.

EfficientNet has been used as a backbone in various applications, including firearms monitoring, litter detection, remote sensing scene classification, and weed detection (Lasloum et al., 2021; Cordova et al., 2022; Chatterjee et al., 2023; Jin et al., 2023). The models have been shown to provide accurate and efficient results in these domains.

## 3 Materials and methods

This research aimed to assess the ability of Mask R-CNN to detect and segment quinoa panicles accurately and efficiently, utilizing various backbone architectures. The study investigated the optimal backbone architecture for Mask R-CNN in detecting and segmenting quinoa panicles under natural field conditions. The main goal was to phenotype distinct quinoa genotypes. The significant contributions of this research are outlined below:

- Building a highly and precisely annotated dataset of quinoa images to train models.
- Studying the phenotyping aspects of quinoa through detection and segmentation of panicles.
- Developing a model for automatic detection and counting panicles despite quinoa’s challenging structure.

**Figure 3:**
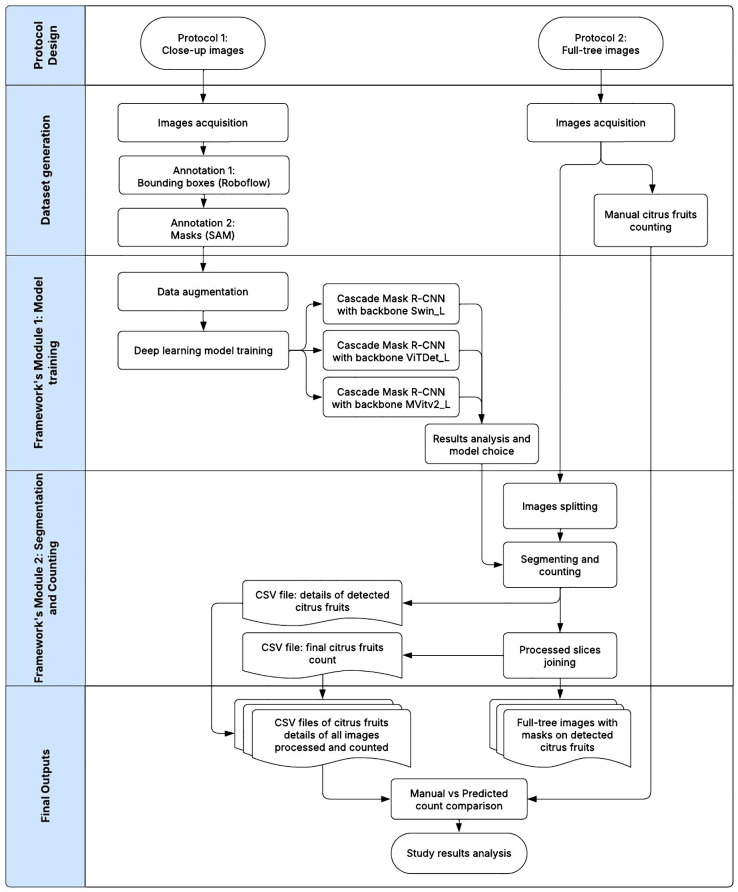
Overall process flowchart of quinoa panicles detection and segmentation.

The following flowchart in **Figure 3** comprehensively illustrates the activities integral to the study. The following sections describe the processes in detail.

### 3.1 Plant materials

The experiment was carried out at the Tassaout research station belonging to INRA (National Institute of Agronomic Research, Morocco, 31° 49’ 12.6768”N, 7° 26’ 32.4096”W) with the aim to understand the behavior of each genotype under two different irrigation protocols: Full Irrigation and Deficit Irrigation. Six genotypes were sown on January 6th, 2022, under a Split-Split Plot Design with a planting density of 2.5 kg/ha. Each plot was 2.5 meters wide and 3 meters high. Seeds were provided by NordGen genebanks: Genotype 5 = Puno Variety 3706, and from IPK:

- Genotype 1= CHEN 144
- Genotype 2 = CHEN 522
- Genotype 3= CHEN 250
- Genotype 4= CHEN 158
- Genotype 6= CHEN 67

### 3.2 Data collection

Throughout the growing season, several phenotyping measurements were conducted. Once the quinoa had reached its full height on 25th April 2022, ground-based images were captured for field phenotyping. The imaging equipment consisted of a Sony ILCE-5100, a 24.3-megapixel digital camera (6000 × 4000 pixels), and a 35mm camera lens attached to a monopod. A viewing angle of 40°from the monopod head was selected to capture the entire plot area with minimal overlap. In addition, the field imagery was captured under natural lighting conditions using a color checker for accuracy. The camera sensor was placed 2.5m above the ground and 1m from the plot’s border. The camera settings were as follows: Focal length: 18 mm, Aperture: f/10.0, ISO: 400, and Exposure time: 1/500 s. The ground resolution of images was approximately between 0.036 and 0.04 cm per pixel.

### 3.3 Data preparation

**Figure 4:**
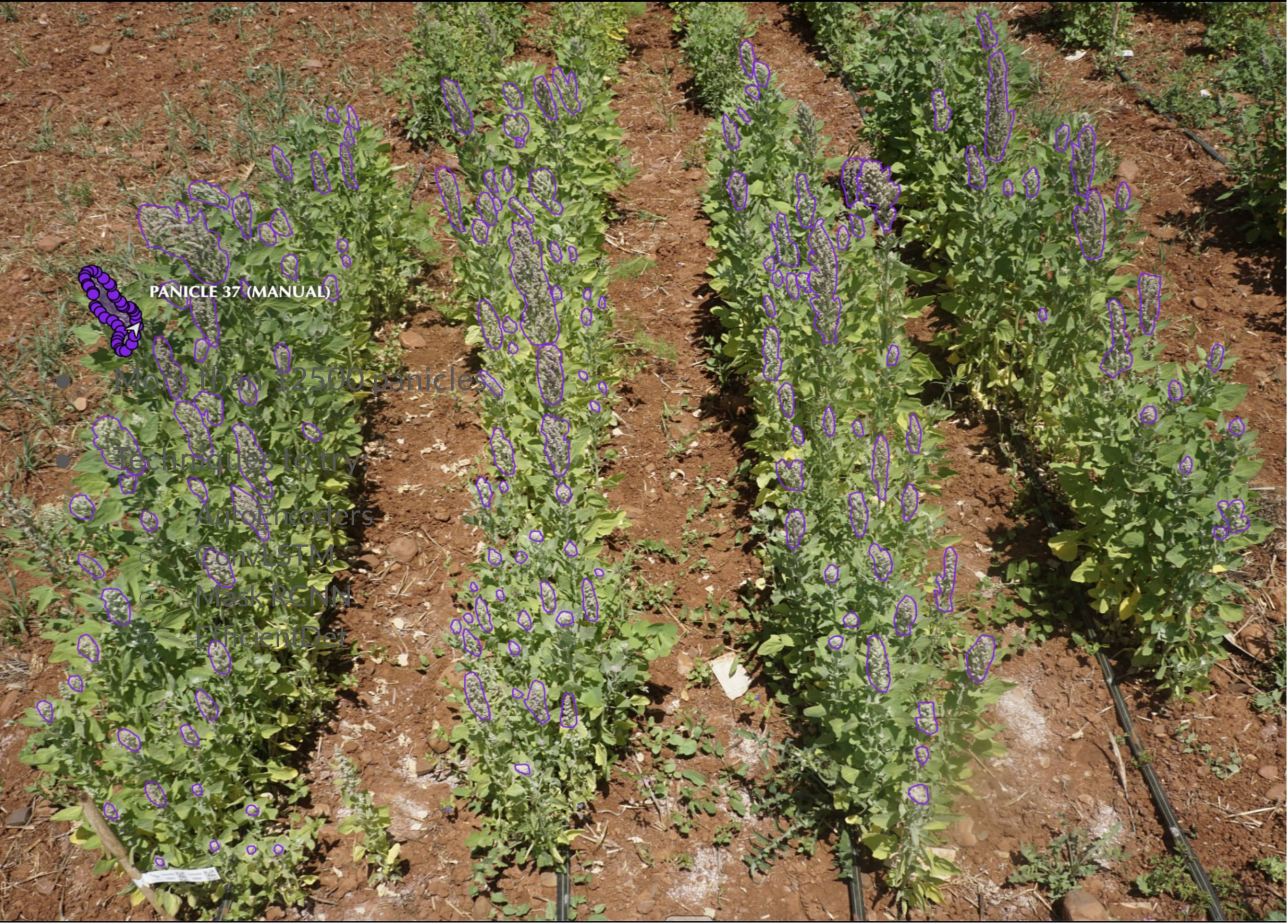
Example of annotation using points to draw polygons on each quinoa panicle.

A total of 288 plots were analyzed (144 per irrigation treatment). On average, three images were captured for each plot, resulting in more than 800 images. Using the Computer Vision Annotation Tool (CVAT) ^2^ and polygon annotation, over 12,500 panicles were manually annotated and saved in COCO format. **Figure 4** presents an example of annotation. The dataset was divided into 8,543 panicles for training, 2,664 for validation, and 1,330 for testing. Another 288 images were selected, different from the training dataset and uniquely presenting each plot, to evaluate the performance of our model against the visual counting of panicles.

### 3.4 Model architecture

We used Mask R-CNN to detect and segment quinoa panicles. We adopted diverse backbones mentioned in section 2.4 and compared the results with actual counting. Finally, our proposal involves enhancing the Mask R-CNN structure by replacing the feature extractor with a combined FPN (Feature Pyramid Network) and an upgraded version of the EfficientNet-B7 backbone. FPN, feature pyramid network (FPN), is a neural network used in computer vision for object detection (Lin et al., 2017). Combining features from multiple levels of a convolutional network is necessary to detect objects of varying sizes to achieve optimal object detection. FPN is a powerful tool that generates high-quality, multi-scale feature maps. It is composed of both a bottom-up and a top-down pathway.

While EfficientNet-B7 can be paired with various activation functions, one of its default choices has been the Swish activation function, which often outperforms traditional activations like ReLU in deeper models by introducing a smoother and adaptive non-linearity. The activation function is crucial in calculating the weighted sum of inputs and biases in deep CNN. It also helps in minimizing errors between the output and the expected value. However, the emergence of the Mish activation function has opened new avenues for enhancing the performance of models like EfficientNet-B7 even further. Mish (Misra, 2020), a novel self-regularized non-monotonic activation function, has demonstrated superior potential to capture a broader spectrum of features and reduce the risk of vanishing gradients, especially in deeper networks. Mish function can be defined as:


f(x)=x∗tanh(softplus(x))

where


$$softplus(x)=\text{ln}(1+e^{x})$$

Knowing the challenging features of panicles, we propose integrating the Mish activation function into the EfficientNet-B7 network. This combination has the potential to enable the network to learn more elaborate representations and better adapt to complex visual scenarios. As a result, it can refine the object detection process to a previously unattainable level.

The backbone network produces hierarchical feature maps. These feature maps are fed into the Region Proposal Network (RPN). The RPN systematically slides over these maps, generating a series of region proposals. These proposals highlight potential bounding boxes that could contain objects. For each of these proposed regions, the RPN also predicts the likelihood of an object’s presence, ensuring that regions with higher probabilities are forwarded for detailed processing. After the RPN, the proposals are passed to a Region of Interest (RoI) Align module. This module warps each proposal to a fixed size, making it feasible for further processing by standard layers. Following the RoI Align, a set of fully connected layers predict class labels and adjust bounding box coordinates for these proposals, refining their positions and sizes.

**Figure 5:**
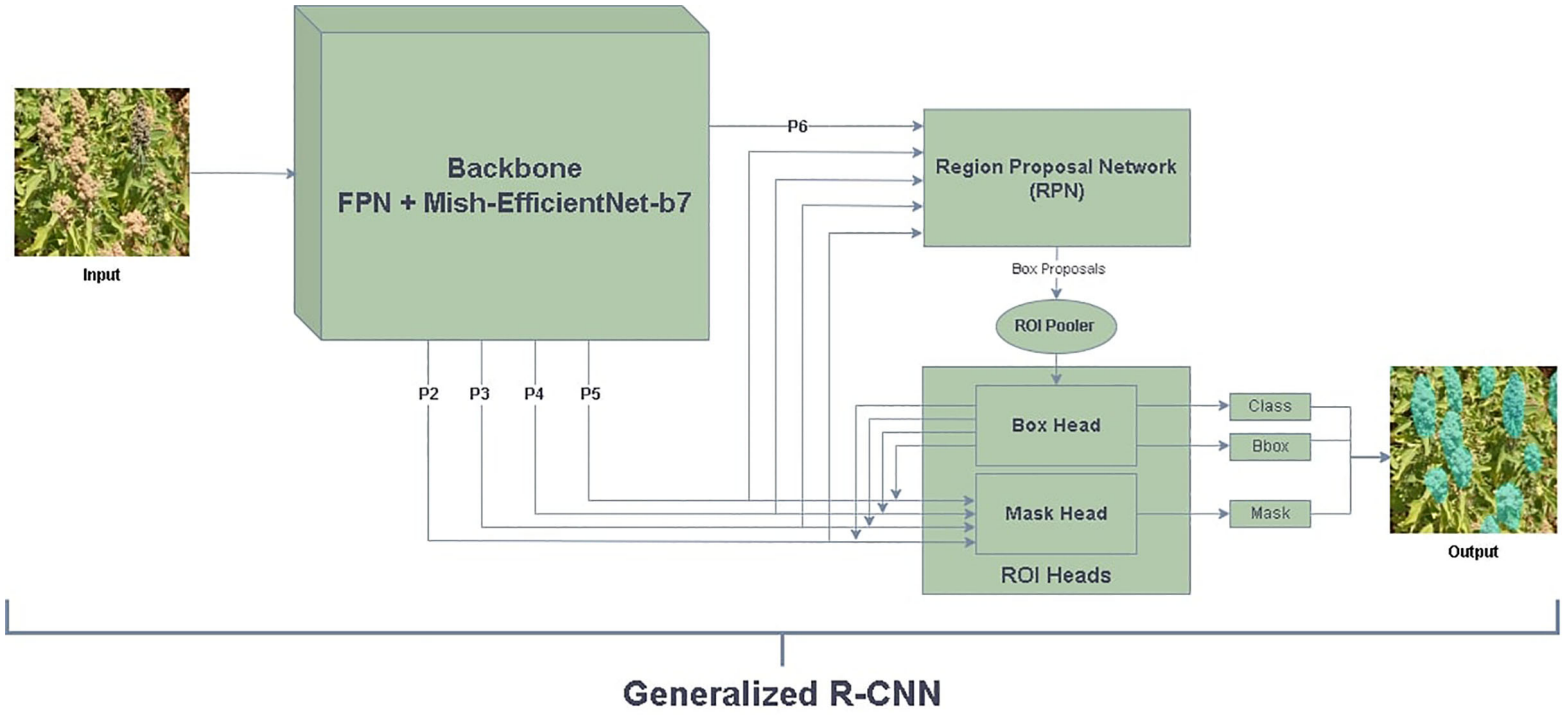
Mask R-CNN based Mish-EfficientNet-B7 and FPN model for panicle instance segmentation.

Parallel to this bounding box regression and classification, Mask R-CNN introduces another branch for mask prediction. Unlike the bounding box prediction, which provides a rectangular region of the object, this mask branch uses a small Fully Convolutional Network (FCN) to produce a binary mask for every class label. The mask corresponds to the precise shape of the object within the bounding box. In this context, for the panicle, the FCN’s role is to delineate the exact contours of the panicle, allowing for its accurate segmentation from the background or other entities in the image. **Figure 5** displays the model’s architecture.

### 3.5 Model training and evaluation

This study was implemented using Python 3.9 and Pytorch 2.0 framework. All the models were trained in Google Colab A100-SXM4-40GB GPU. The Mask R-CNN model is implemented using detectron2, a powerful software system developed by Facebook AI Research (FAIR) (Wu et al., 2019). Detectron2 is an upgraded version of Detectron, coded in PyTorch with a more modular design. It can implement advanced algorithms such as Faster R-CNN, Mask R-CNN, RetinaNet, and DensePose. Its heightened flexibility and extensibility have made it FAIR’s most popular open-source project. After some tests, the model was trained to 4000 iterations.

Due to the limited availability of datasets, transfer learning has become a popular approach to train deep learning models more efficiently and stably (Szegedy et al., 2015). By leveraging pre-trained CNN features from ImageNet, which consists of 1000 object categories and 1.2 million images, state-of-the-art results have been achieved in various image processing tasks, ranging from image classification to image captioning. To our knowledge, it is highly improbable that images of quinoa panicles are in the ImageNet dataset or other public datasets, given that quinoa is not a widely researched crop. To address this, fine-tuning the pre-trained model’s layers with our labeled panicle image is necessary.

Data augmentation is necessary to improve the dataset for training, as it increases the number of images while maintaining quality (Perez and Wang, 2017). We applied data augmentation using the defined functions:

- RandomApply: Randomly apply an augmentation with a given probability.
- RandomFlip: Flip the image horizontally or vertically with the given probability.
- ResizeShortestEdge: Resize the image while keeping the aspect ratio unchanged.
- RandomCrop: Randomly crop a rectangle region out of an image.

Hyperparameters play a pivotal role in the training and performance of deep learning models, and Mask R-CNN is no exception. In Mask R-CNN, hyperparameters, such as learning rate, batch size, weight decay, and anchor scales, significantly influence the network’s convergence rate, its adaptability to the dataset, and its detection and segmentation accuracy. Several hyperparameters were fine-tuned in the experiments to better align with our dataset characteristics.

Since we have a limited-size dataset, we set the normalization for the conv layers in Box Head and Mask Head to “GN” (Group Normalization) (Wu and He, 2018). We did the same for FPN and chose the Group Normalization instead of Layer Normalization. The learning rate was set to 4e-05, with a weight decay of 0.05 and AdamW (Loshchilov and Hutter, 2019) as the optimization method.

Mask R-CNN introduces a novel loss function for the mask branch, the binary cross-entropy loss. This is incorporated alongside the existing losses - the softmax loss for class labels and the smooth L1 loss for bounding box coordinates. These individual losses are summed up to obtain the final loss function (L) of the Mask R-CNN model, represented mathematically as follows (He et al., 2017):


$$L=L_{cls}+L_{box}+L_{mask}$$

Where $L_{cls}$ is the log loss over two classes (object vs. not object), $L_{box}$ is the smooth L1 loss for the bounding box regression, and $L_{mask}$ is the average binary cross-entropy loss.

We analyzed the predicted segmentation masks in the output images obtained from the trained Mask R-CNN. The aim was to evaluate the effect of the different backbone parts in the Mask R-CNN mask. This analysis used two metrics: average precision (AP) and IoU. IoU is a crucial metric used to assess segmentation models (Zhou et al., 2019), commonly referred to as Jaccard’s Index. This metric quantifies how effectively the model can distinguish objects from their backgrounds in an image. IoU is widely used in several computer vision applications, including autonomous vehicles, security systems, and medical imaging.

The IoU between the ground-truth panicle region, $A_{gt}$ , and the predicted panicle region, $A_{p}$ , was calculated as follows:


$$IoU(A_{gt,Ap})=\frac{A_{gt}\cap A_{p}}{A_{gt}\cup A_{p}}$$

In order to assess the performance of our models, we will employ the official COCO evaluation metrics in Python, including AP50 defined as AP at IoU = 0.5 (AP50). This version of the AP metric evaluates average precision when the Intersection over Union (IoU) threshold is set at 0.5. A higher IoU threshold means stricter evaluation criteria and an IoU of 0.5 is commonly used for many detection tasks.

These metrics offer a thorough evaluation of bounding box and mask annotations. However, when evaluating quinoa, we must consider the possibility of additional panicles from lateral branches that do not contribute to the yield (Stanschewski et al., 2021). To guarantee the accuracy of our predicted count, we cross-referenced it with the count determined by an expert. The models may then be assessed more accurately by utilizing both approaches.

## 4 Results

### 4.1 Model training results

**Figure 6:**
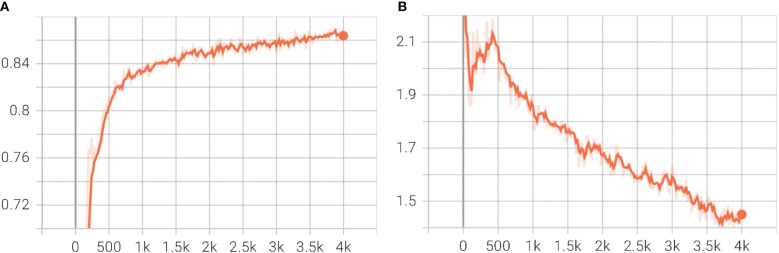
Plot of Mask R CNN training accuracy and Total Loss during training with iterations steps. **(A)** Training Accuracy; **(B)** Total Loss.

The total loss and training accuracy were considered in assessing the Mask R-CNN model’s training. **Figures 6A, B** depict the Mask R-CNN training accuracy and total loss with the number of iterations, respectively. At the completion of 4,000 iterations, the training accuracy was determined to be 86.3%, and the Total Loss was 1.42. Throughout the training, the predicted masks over objects were compared to the ground truth data after a specified number of iterations, enabling the calculation of training accuracy for the Mask R-CNN model.

**Figure 7:**
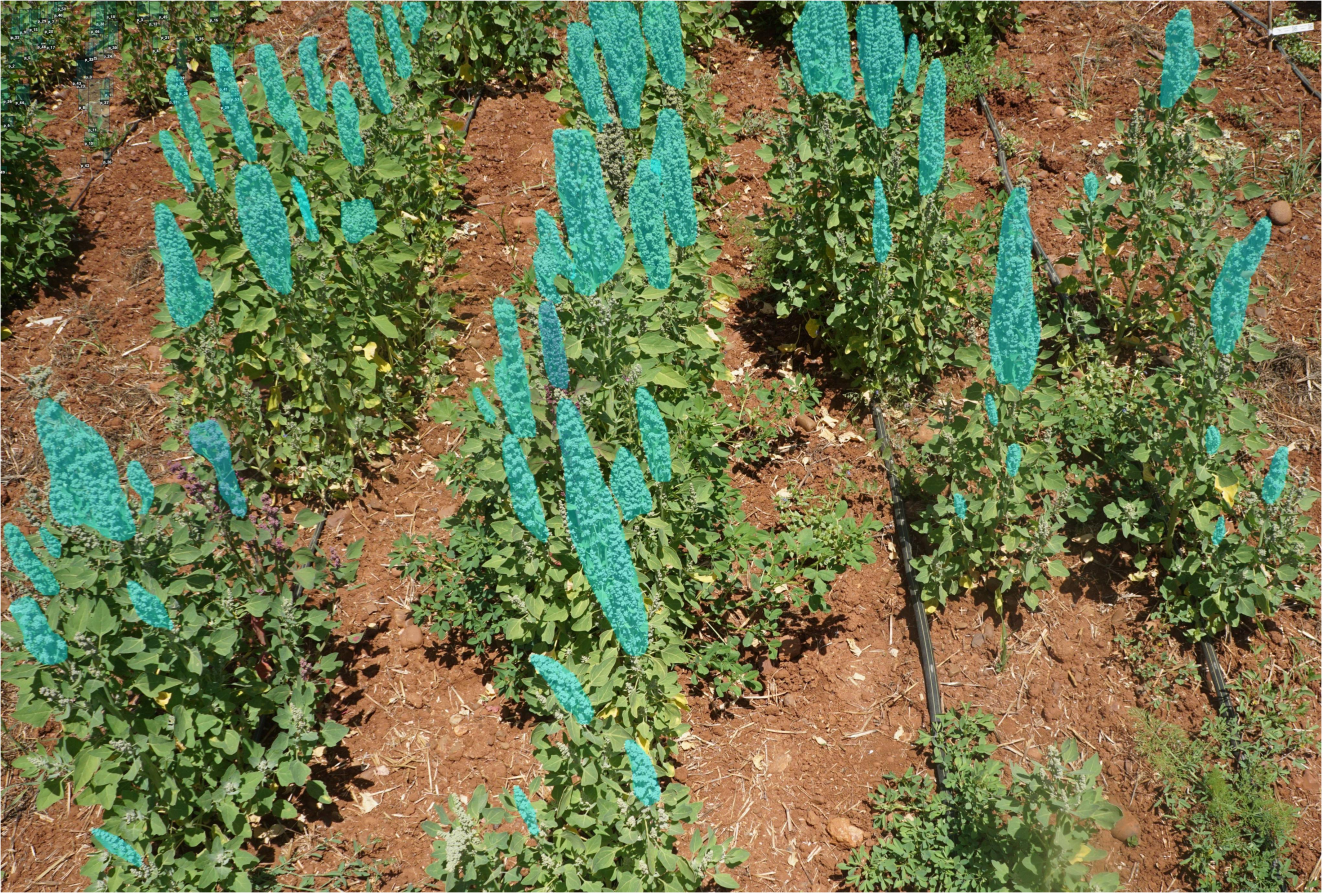
Predicted masks on detected panicles using our proposed model.

Upon completion of model training, we evaluated the models using the test dataset, and CSV files were produced to showcase the location of each panicle in the image and their respective mask sizes. Our predictors operate under two defined thresholds: 0.5 and 0.7. The image depicted in **Figure 7** exemplifies the prediction abilities of the MishEfficientNet-based Mask R-CNN model under the 0.5 threshold. For an IOU of 0.5, the model achieves an AP of 50.632 for bounding box annotation and 50.773 for mask annotation. At a higher IOU threshold of 0.7, the model’s performance decreases, recording an AP of 44.391 for bounding box annotation and 44.588 for mask annotation. This indicates that while the model performs reasonably well at a lower IOU threshold, its performance declines as the threshold increases, reflecting the challenges in precise object detection and segmentation of panicles.

### 4.2 Comparison of backbones

To ensure a thorough analysis of our proposed model’s performance, we utilized the Mask R-CNN architecture with diverse backbones. While the ResNet backbone is the standard in the original Mask RCNN, we conducted an in-depth comparison by incorporating other top-performing backbones mentioned in section 2.4 to evaluate how various feature extraction methods impact instance segmentation and object detection tasks. To ensure unbiased evaluations, we attempted to train all versions of the models using the same dataset and conditions. However, due to differences in backbone structures, we adjusted the learning rate accordingly to maintain the integrity of our comparisons.

To further evaluate the effectiveness of the Mish activation function, we compared our proposed methodology with the original EfficientNet-B7 network that uses the Swish activation function.

**Figure 8:**
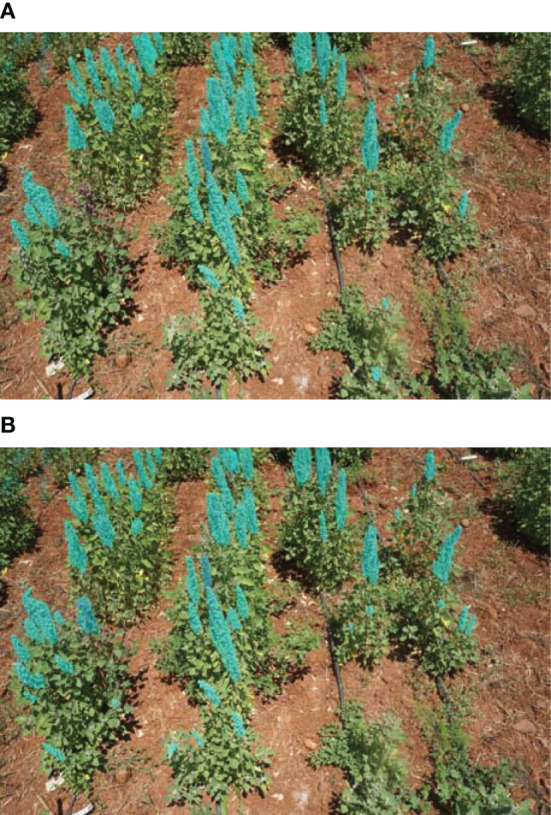
Comparison between predicted masks of the two activation functions: Swish and Mish. **(A)** Swish-EfficientNet-B7, **(B)** Proposed Model’s Predictions.

When using the Mish activation function, the model achieves an AP of 50.632 for bounding box annotation and 50.773 for mask annotation. On the other hand, the Swish activation function yields an AP of 48.928 for bounding box annotation and 49.581 for mask annotation. These results indicate that the Mish-EfficientNet-B7 backbone performs better than the Swish-EfficientNet-B7 backbone in terms of both object detection and segmentation at an IOU threshold of 0.5, highlighting the effectiveness of the Mish activation function in improving model accuracy. It is clear from the results presented in **Figure 8A** that there are some repetitions in detection (from the mask color), compared to our proposed model’s results in **Figure 8B**, proving the higher performance of the Mish function.

Finally, we compared the results of the rest of the backbones mentioned in section 2.4 with our proposed model. The **Table 2** summarizes the comparative analysis of the different backbones evaluated with the test dataset using two thresholds of detection probabilities 50% and 70%.

**Table 2:** Results of the AP50 metric in the comparative study using the thresholds 0.5 and 0.7.

Backbone	Threshold 0.5		Threshold 0.7	
	Bbox %	Mask %	Bbox %	Mask %
ResNet50	45.299	44.456	38.254	38.209
ResNet101	44.034	46.073	38.852	41.490
ViTDet_b	46.097	46.846	37.330	37.207
Swin_b	27.106	26.934	15.161	15.078
Mish-based EfficientNet_B7 + FPN(LN)	46.186	47.405	41.687	42.138
Mish-based EfficientNet_B7 + FPN(GN)	50.632	50.773	44.391	44.588

**Figure 9:**
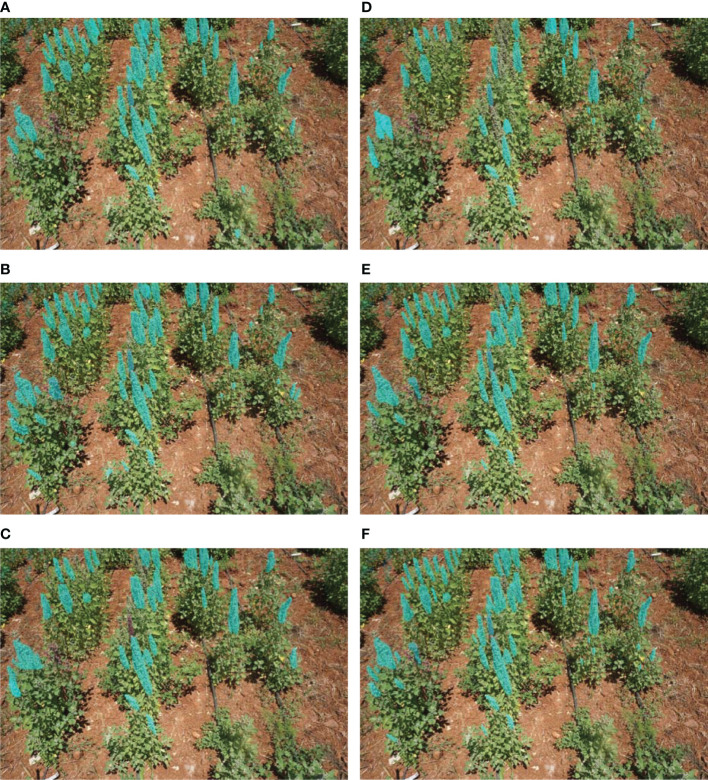
Comparison between resulted masks in different methods. **(A)** ResNet50, **(B)** ResNet101, **(C)** ViTDet, **(D)** Swin, **(E)** MishEfficientNet-B7 + FPN(LN), **(F)** MishEfficientNet-B7 + FPN(GN).

The quinoa panicles prediction results of the different backbones used in the comparative study with a 50% threshold are presented in **Figure 9**.

### 4.3 Counting analysis

**Figure 10:**
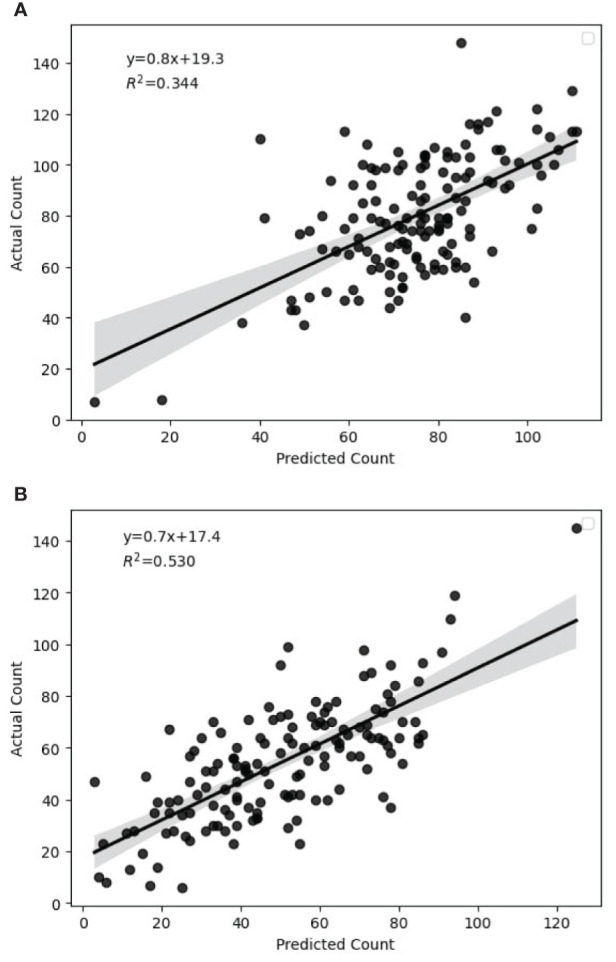
Correlation between predicted and actual count in full and deficit irrigations. **(A)** Full Irrigation, **(B)** Deficit Irrigation.

In this research study, the assessment of methods involved the solicitation of expert knowledge in the counting of primary quinoa panicles that contribute to the overall yield. Subsequently, the predicted counts were compared to the actual counts for each image, in order to evaluate the accuracy of the counting method. **Figures 10A, B** present the relationship between the ground truth number of panicles and the estimated number of panicles across all genotypes for full and deficit irrigation, respectively. The regression statistics for the predicted versus actual values of panicle counts offer valuable insights into the model’s performance. **Table 3** summarizes the regression statistics in full and deficit irrigation, where Multiple R represents the multiple correlation coefficient between actual and predicted count.

**Table 3:** Summary output of regression statistics in full and deficit irrigation.

Regression Performance Metrics	Full Irrigation	Deficit Irrigation
Multiple R	0.586	0.727
R Square	0.344	0.529
Adjusted R Square	0.339	0.526
Standard Error	13.756	15.477

**Figure 11:**
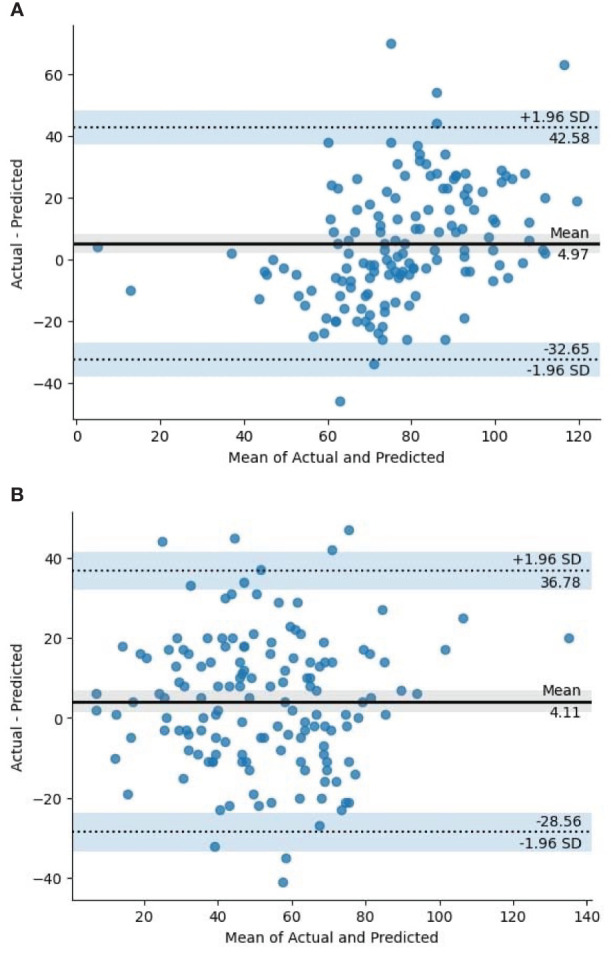
Bland-Altman plot in full and deficit irrigations. **(A)** Full Irrigation, **(B)** Deficit Irrigation.

For further analysis, we used the Bland-Altman plot as presented in **Figure 11**. The Bland-Altman plot is a pivotal analytical tool in evaluating the performance of our model designed to count panicles, facilitating a deep understanding of the discrepancies between predicted and actual counts. It graphically represents the agreement between two quantitative measurements by plotting the difference against their average. Our model enables a meticulous inspection of the systemic differences (biases) and random errors inherent in the model’s predictions compared to the actual panicle counts, providing insights that can drive model refinements and optimizations. **Figures 11A, B** show the Bland-Altman plot for full and deficit irrigation, respectively.

To understand the variability in the results, we made regression analyses on each genotype. **Tables 4**, **5** present a summary of analyses for each genotype in full and deficit irrigation, respectively.

**Table 4:** Summary output of regression statistics in Full Irrigation for all genotypes.

Regression Performance Metrics	Genotype 1	Genotype 2	Genotype 3	Genotype 4	Genotype 5	Genotype 6
Multiple R	0.603	0.331	0.343	0.863	0.767	0.352
R Square	0.364	0.110	0.117	0.744	0.588	0.124
Adjusted R Square	0.335	0.069	0.077	0.733	0.569	0.084
Standard Error	17.081	20.508	23.108	14.846	15.960	20.005

**Table 5:** Summary output of regression statistics in Deficit Irrigation for all genotypes.

Regression Performance Metrics	Genotype 1	Genotype 2	Genotype 3	Genotype 4	Genotype 5	Genotype 6
Multiple R	0.796	0.520	0.797	0.614	0.725	0.796
R Square	0.634	0.270	0.636	0.378	0.526	0.635
Adjusted R Square	0.617	0.237	0.620	0.349	0.505	0.618
Standard Error	17.238	17.479	13.001	18.030	16.546	11.598

## 5 Discussion

### 5.1 Algorithmic level

It has been demonstrated by this study that deep learning techniques can be used to detect quinoa panicles of six different genotypes despite the complexity of the selected trait and the variability of the panicle (type, size, color, and density).

The backbone architecture heavily influences the performance of Mask R-CNN models. We comprehensively evaluated various backbones, including ResNet50, ResNet101, ViTDet, Swin, and Mish-based EfficientNet B7, with LN as the default normalization layer in FPN. The proposed method, which utilizes Mish-based EfficientNet B7 with GN instead of LN, produced the best results, as shown in **Figure 9**. This outcome points to the unique architectural design and training strategy as the key factors in the model’s performance.

Although ResNet50 and ResNet101 have proven effective in multiple applications, their limited receptive field and lack of attention mechanisms may impede their performance. ViTDet, on the other hand, employs a transformer-based architecture that can more efficiently capture long-range dependencies by processing input images as a sequence of patches. However, its computation costs may surpass those of other backbones due to the self-attention mechanism. Additionally, accurately extracting features from the shape and structure of panicles has posed a challenge.

In the expansive realm of agricultural research and technology, it is surprising to note that there is a significant gap in the literature about the instance segmentation and counting of quinoa panicles. Mask R-CNN has been, however, has been used extensively in other crops like wheat. In (Su et al., 2020), for instance, the authors used Mask R-CNN with ResNet-101+FPN backbone, and the AP50 values for detection and segmentation of wheat spikes were 56.69% and 57.16%, respectively. An improved version of Mask R-CNN was used to detect and segment grape clusters in the field based on the attention mechanism and ResNet50 + FPN backbone in the study of (Shen et al., 2022). They achieved high AP50 values, with 85.60% and 87.10%, respectively, in detection and segmentation.

Among the tested backbones, EfficientNet-B7 with Mish activation and Group Normalization demonstrated the highest segmentation accuracy and robustness, making it the most suitable choice for quinoa panicle detection in challenging field conditions.

### 5.2 Experimental level

During the evaluation, our model demonstrated high performance with exceptional AP values. Nevertheless, we observed a discrepancy between the actual and predicted count. The regression results offer an intriguing perspective on the predictive capability of our model for panicle counts. Given that these insights are derived from 288 observations, the dataset’s size provides reasonable confidence in the results. **Table 4** shows that the application of the model on Genotype 4 seems to be the most effective in terms of fit and predictive power, followed by Genotype 5. The other genotypes, especially Genotypes 2, 3, and 6, demonstrate weaker predictive capabilities based on the presented metrics.

The variation in R^2^ values among the six quinoa genotypes is attributed to a combination of genetic, environmental, or experimental factors. Upon closer examination, we found that this correlation was influenced by the type of the panicle for each genotype. For example, for Genotype 5 (Variety Puno), the R square did not change in full and deficit irrigation, which means that this genotype was not statistically influenced by the stress level we applied. This result is in concordance with the provided information on the Puno variety, which has been registered as a new quinoa variety in Europe, bred from Chilean and Peruvian landraces and selected for earliness, lower height at harvest and adaptation to Mediterranean conditions (Lavini et al., 2014).

For Genotype 4, the significant drop in R square value from 0.74 under full irrigation to 0.37 during deficit irrigation highlights the considerable impact of water availability on its panicle structure, particularly in terms of density. The changes made to the panicle structure caused the model to struggle with detection and segmentation, leading to a decrease in the R square. Originating from Bolivia, this genotype, cataloged at the IPK with the accession code N°CHEN 158, exhibits a unique sensitivity to water stress. This characteristic is intrinsically linked to its genetic and geographical origins. The genotype’s native environment in Bolivia, known for its varied climatic conditions, has likely influenced the development of specific traits in this plant. Under water stress conditions, it exhibits a marked panicle size and density reduction.

This study’s main finding is the genotypes’ sensitivity to water availability, particularly for Genotypes 3, 4, and 6, demonstrating a pronounced variation in panicle structure and density in response to irrigation levels, underscoring the significance of understanding genotype-specific responses in agricultural practices. Understanding these links is crucial for developing effective irrigation strategies and selecting genotypes best suited for cultivation in water-limited environments.

Many authors described that some quinoa genotypes might exhibit higher phenotypic plasticity, which is the ability of an organism to change its phenotype in response to environmental conditions (Mhada et al., 2014; Becker et al., 2017; del Pozo et al., 2023). Genotypes with higher plasticity may have more consistent responses across various conditions, leading to higher R² values. The other genotypes exhibited an increase in R square in deficit irrigation compared to full irrigation. This can be explained by the fact that when sown in arid lands, their panicles get denser but smaller, helping in better detection using the model. The observed reduction in panicle size is similar to what was found when quinoa was grown under drought or saline conditions (Maliro et al., 2017).

### 5.3 Limitations and future perspectives

It has been observed that Mask R-CNN is a widely used method for detecting and segmenting crops. However, the accuracy of the model depends mainly on the quality of the data, such as the size of the dataset, image-taking protocols, resolution, etc. Although the current model can predict panicle count to some extent, there is still much scope for improvement to enhance its predictive abilities. Moreover, acquiring a large image library of quinoa is challenging, as it is an understudied crop with a shortage of available datasets. Our study’s dataset was relatively small compared to the datasets available in the literature for other crops, such as wheat.

Quinoa’s unique morphology presents a challenge when utilizing deep learning models. Phenotyping quinoa requires a precise image capture and model selection protocol, as each genotype behaves differently. The differences between genotypes regarding their phenological stages can significantly impact the model’s accuracy, as their responses at specific observational times may vary. This should be taken into account when taking pictures for panicle counting. Additionally, standardizing image capture through consistent camera angles, controlled lighting, and calibration tools can enhance data uniformity and improve model generalization.

Finally, with the rapid and exponential evolution of computer vision technologies, there is an undeniable potential for groundbreaking advancements in panicle detection. As these models continue to mature, harnessing the capabilities of the latest cutting-edge algorithms can significantly enhance the accuracy, efficiency, and speed of panicle detection processes. Such advancements could revolutionize how researchers approach crop management, leading to optimized yields and more sustainable agricultural practices.

Future research should consider integrating this instance segmentation approach with unmanned aerial vehicles (UAVs) for large-scale field monitoring, as well as with IoT-based environmental sensors for real-time crop condition tracking. By combining these tools into a cohesive decision-support system, we could greatly enhance the precision and scalability of advanced farming practices for quinoa and other emerging crops.

## 6 Conclusion

In modern agricultural research, instance segmentation is an indispensable tool for enhancing the accuracy and precision of crop analysis. Its role in the study of quinoa, a crop with substantial nutritional value, is particularly significant. By providing nuanced information about each quinoa panicle, instance segmentation enables researchers to distinguish it from the intricate background and other overlapping entities. This level of detail is invaluable in evaluating factors such as health, growth, and yield estimations.

The Mask R-CNN algorithm is a highly effective tool for performing instance segmentation. For quinoa specifically, Mask R-CNN offers impressive accuracy by identifying panicles and generating precise masks ideal for more in-depth analysis. This feature can be especially valuable for conducting phenotype analyses and detecting anomalies or illnesses.

The backbone networks in Mask R-CNN, essentially the convolutional base layers, play a crucial role in feature extraction. Their significance cannot be overstated. They dictate the quality of features extracted from the image before the region proposal and segmentation processes commence. The backbone choice often impacts the Mask R-CNN’s precision, speed, and overall performance. Given quinoa’s unique structure and texture, an appropriate backbone can optimize segmentation accuracy.

The intricate composition of quinoa presents a noteworthy hurdle when utilizing Mask R-CNN. The panicles of quinoa showcase a range of differences regarding their dimensions, contours, and tones, with some discrepancies barely noticeable. Moreover, the tightly clustered configuration of the panicles, coupled with potential overlap, can obstruct Mask R-CNN’s ability to segment each occurrence precisely. These obstacles underscore the importance of refining the model or introducing supplementary pre-processing measures to augment the detection’s durability.

Our research sought to assess different frameworks for Mask R-CNN and introduce a novel approach employing an upgraded EfficientNet version. Our foremost goal is to boost the precision of feature extraction to gain deeper insights into quinoa panicles. This will facilitate the examination of several phenotyping factors, such as yield estimation. Our proposed methodology involves applying EfficientNetB7 with Mish activation function and combined with FPN and Group Normalization. Our methodology successfully outperformed the other backbones in this study.

In conclusion, Mask R-CNN is widely used in segmentation but still faces challenges when used in field images, especially for crops such as quinoa. In addition, while instance segmentation and Mask R-CNN specifically hold promise for transforming quinoa analysis, addressing the accompanying obstacles and constraints is imperative. Such efforts will facilitate the creation of more sophisticated models that can better account for the intricacies of quinoa.

This work contributes directly to Sustainable Development Goal 2: Zero Hunger by promoting the use of AI in agriculture. AI’s potential in enhancing crop monitoring, precision breeding, and productivity under challenging conditions is immense. Our approach, which enables automated, high-precision phenotyping of stress-resilient crops like quinoa, is a powerful tool in addressing food insecurity in saline and arid regions.

## Data Availability

The raw data supporting the conclusions of this article will be made available by the authors, without undue reservation.

## Footnotes

1 FAO (2013). International Year of Quinoa: A Future Sown Thousands of Years Ago. Retrieved from http://www.fao.org/quinoa-2013/en/.

2 https://github.com/opencv/cvat
