# Correction: Optimizing Mask R-CNN for enhanced quinoa panicle detection and segmentation in precision agriculture

**DOI:** 10.3389/fpls.2025.1664228

**Published:** 2025-08-01

**Authors:** Manal El Akrouchi, Manal Mhada, Dachena Romain Gracia, Malcolm J. Hawkesford, Bruno Gérard

**Affiliations:** ^1^ College of Agriculture and Environmental Sciences, University Mohammed VI Polytechnic (UM6P), Ben Guerir, Morocco; ^2^ School of Collective Intelligence, University Mohammed VI Polytechnic (UM6P), Rabat, Morocco; ^3^ Sustainable Soils and Crops Department, Rothamsted Research, Harpenden, Hertfordshire, United Kingdom

**Keywords:** Mask R-CNN, instance segmentation, quinoa, precision agriculture, deep learning

There was a mistake in **Figure 3** as published. I was working on two papers simultaneously, this one and another related to citrus (see this link). While preparing the flowcharts for both projects, I inadvertently used the same name for both files, which led to this confusion. The corrected **Figure 3** appears below.

**Figure 3:**
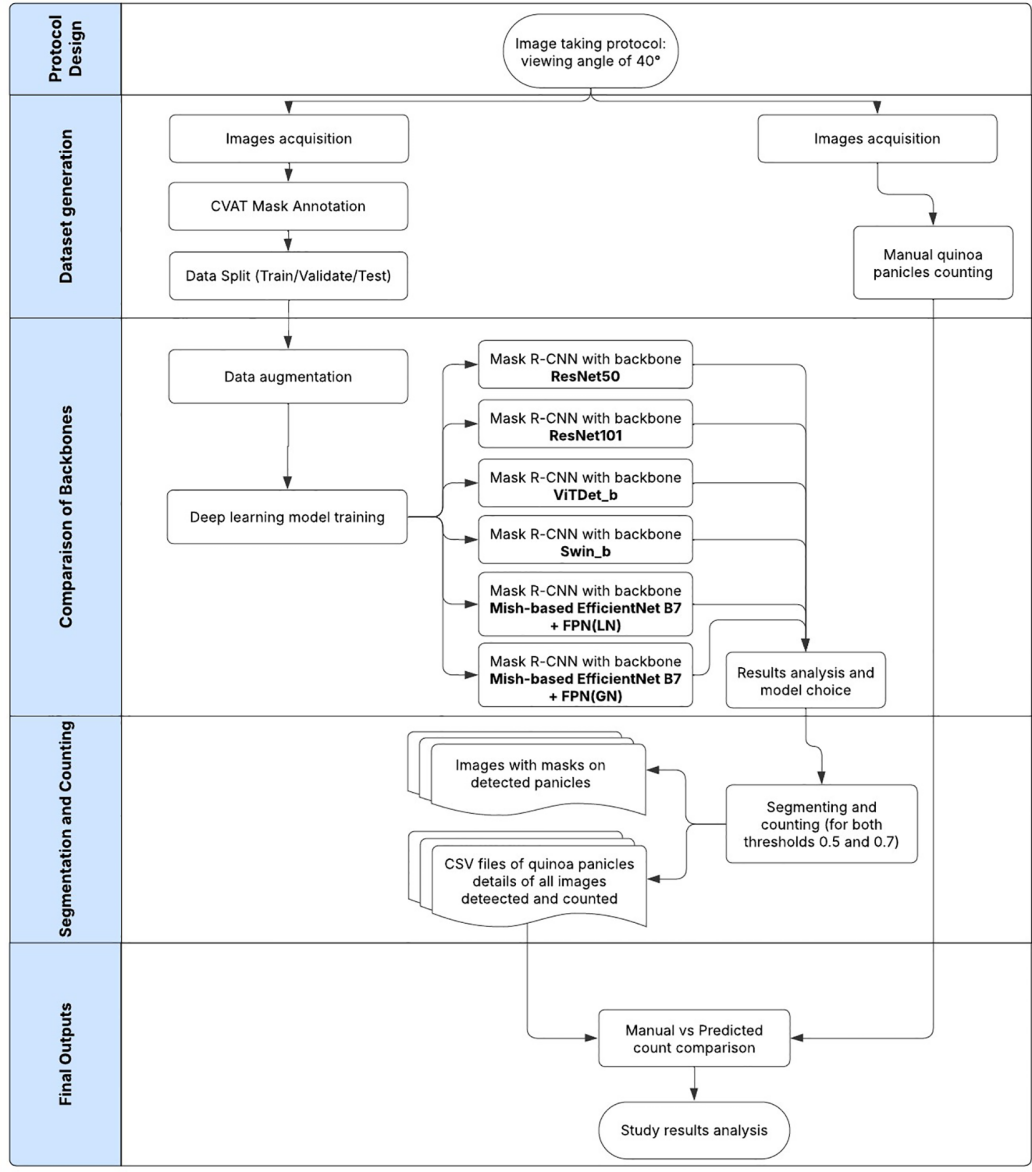
Overall process flowchart of quinoa panicles detection and segmentation.

The original version of this article has been updated.

